# The Impact Mechanism of Entrepreneurial Team Expertise Heterogeneity on Entrepreneurial Decision

**DOI:** 10.3389/fpsyg.2021.732857

**Published:** 2021-10-04

**Authors:** Suyang Ye, Yunchun Xiao, Bin Yang, Dong Zhang

**Affiliations:** ^1^Department of Human Resource Management, School of Business Administration, Zhejiang Gongshang University, Hangzhou, China; ^2^Zhejiang Tianyuan Building Material Company, Hangzhou, China

**Keywords:** entrepreneurial team expertise heterogeneity, team knowledge integration, entrepreneurial decision-making, team reflexivity, information processing theory

## Abstract

Under the background of economic globalization and industrial revolution, team entrepreneurship has drawn increased attention. Team entrepreneurship is considered to be advantageous in its capability of integrating more abundant resources and of sharing knowledge, information, and complementary skills among members of the entrepreneurial team, enabling entrepreneurial enterprises to successfully adapt to the highly uncertain entrepreneurial environment. In recent years, the relationship between the heterogeneity of the entrepreneurial team and its decision-making quality has attracted much attention in the management research field. However, the research results on such topic are quite inconsistent. Based on the information processing theory, the greater the market uncertainty the entrepreneurial team faces, the stronger information integration ability a team will be required to possess. This study investigates the mechanism and boundary conditions of the impact of expertise heterogeneity of the entrepreneurial team on entrepreneurial decision-making. It points out that team knowledge integration and team reflexivity have significant impacts on the relationship between entrepreneurial team expertise heterogeneity and entrepreneurial decision-making. This study adopts the multi-source design approach and collects data from 419 academic entrepreneurial teams in the University Science and Technology Park in Zhejiang Province. Hierarchical regression and bootstrapping methods are also employed for data analysis. The results show that team knowledge integration mediates the relationship between entrepreneurial team expertise heterogeneity and entrepreneurial decision-making, whereas team reflexivity moderates the relationship between entrepreneurial team expertise heterogeneity and team knowledge integration. In the final part, the practical implications for entrepreneurial team are discussed.

## Introduction

Peter F. Drucker used the term “entrepreneurial economy” to describe the present market environment. According to his definition, the entrepreneurial economy is a new economic sociology developed by the country and the society to construct and develop new visions, promote the innovation and development of small- and medium-sized startups, and boost the development of the national economy and the society (Schierjott et al., 2018). In addition, the wide range of new technologies in the information age have also brought more entrepreneurial opportunities to all walks of life, guiding entrepreneurial activities into a brand-new era of development with fast paces. Therefore, the research on entrepreneurial team has become one of the most significant research topics in academic and business circles. Numerous studies show that at present, team entrepreneurship coexists with individual entrepreneurship and has become the main body of entrepreneurship in the present economic society (Cooney, 2005). Other studies also show that the performance and success rate of team entrepreneurship are significantly higher than individual entrepreneurship (Francis and Sandberg, 2000; Townsend et al., 2018).

Most of the previous studies on entrepreneurial team take entrepreneurial success or performance as the outcome variables. However, the analysis on entrepreneurial activities cannot merely be focused on their results while ignoring their processes. In reality, the entrepreneurial decision-making is often regarded as an important indicator of entrepreneurial activities. More specifically, the ability of entrepreneurial teams to make optimal entrepreneurial decisions is generally considered as the key to the success of entrepreneurial activities (Camuffo et al., 2020). In view of the significance of entrepreneurial decision-making, an increasing number of scholars call for the conducting of more research on its generation mechanism and boundary conditions (Engel et al., 2017; Laskovaia et al., 2019). As an important aspect of entrepreneurial team, staff composition in startup enterprises, and the relationship between team heterogeneity and entrepreneurial decision-making, which is an important part of team composition have become a major focus among scholars, especially in recent years (Jin et al., 2017; Zhou et al., 2017, Lazar et al., 2020). The composition heterogeneity of entrepreneurial team is supposed to enhance the decision-making level of entrepreneurial team by improving the team's knowledge resource pool (Bell et al., 2018; Sherf et al., 2018), but some studies have shown that the role of team heterogeneity is not always positive (Tsai and Hsu, 2019). For some teams, the differences of ideas and values brought by background heterogeneity will increase the inter-team conflict, reduce team cohesiveness, and affect the operation efficiency of the team (Hong et al., 2019). This implies that the impact of team heterogeneity on entrepreneurial decision-making may be inconsistent. Therefore, the key to investigate the relationship between team heterogeneity and entrepreneurial decision-making of entrepreneurial team is to explore the mediating and moderation mechanism between the two variables. However, few existing research studies have paid sufficient attention to this problem.

In terms of the mechanism of team composition on entrepreneurial decision-making, previous studies mainly focused on team affective factors, such as team cohesiveness and team positive emotions. However, it remains unknown on how the team integrates the unique knowledge resources of its members. Based on the information process theory and from the perspective of team knowledge integration, this paper aims to reveal the mechanism of entrepreneurial team expertise heterogeneity on team decision-making. According to the information process theory, the greater the market uncertainty is faced by the entrepreneurial team, the stronger information integration ability will a team be required to possess (Tushman and Nadler, 1978). Furthermore, the stronger the information integration ability is which is possessed by the entrepreneurial team, the more effectively the team will be in making high-quality decisions. Therefore, in order to achieve effective entrepreneurial decision-making, an entrepreneurial team is required to effectively integrate and utilize the unique knowledge resources of each team member. From the perspective of team knowledge integration, this study investigates whether the expertise heterogeneity of the entrepreneurial team can promote its decision-making results through effective team knowledge integration.

In addition, studies have shown that when team members are willing to publicly reflect on their working styles and adjust to the changing situations, their different views and opinions can be more effectively integrated (Wang et al., 2021). Furthermore, it is believed in this paper that entrepreneurial teams with distinct team reflexivity also reflect different cooperative levels in the face of expertise heterogeneity among team members. On the one hand, the team with higher reflexivity is more able to analyze specialty and knowledge of each member, assign corresponding job responsibilities, and enhance its knowledge integration ability; on the other hand, the team with lower reflexivity is more prone to habitual behaviors and thinking models (Sherf et al., 2018). Even if the team has unique and rich knowledge resources, it cannot make full use of or integrate the unique resources of members in an entrepreneurial team, thereby weakening its knowledge integration ability. Therefore, according to the relationship between team knowledge integration and team reflexivity, this paper further proposes an integrated model, in which team knowledge integration is believed to be able to mediate the relationship between entrepreneurial team expertise heterogeneity and entrepreneurial decision-making, whereas team reflexivity is believed to be able to moderate the mediating effect.

Two main contributions are expected to be made by this study to the current literature. First, it will shed light on the information process theory by highlighting the role of team knowledge integration as a critical mechanism through which entrepreneurial team expertise heterogeneity positively influences entrepreneurial decision-making. Accordingly, new insights will be added to the current literature focused on entrepreneurial team diversity. More specifically, in addition to taking the team emotional process (i.e., team conflict and team trust) in consideration, the extent of team knowledge integration also matters for the explanation of team expertise heterogeneity's effect. Second, this study adds nuance to its claims by investigating boundary conditions when entrepreneurial team expertise heterogeneity is beneficial. Specifically, we clarified how the interaction between entrepreneurial team expertise heterogeneity and team reflexivity influences team knowledge integration, and therefore, entrepreneurial decision-making. On this basis, we contribute to the understanding of the conditional boundary of entrepreneurial team expertise heterogeneity, which is conducive to the utilization of team knowledge to achieve more effective entrepreneurial decision-making. Finally, this paper answers the important question on how entrepreneurial teams should integrate and utilize unique knowledge resources of their members to make effective entrepreneurial decisions.

## Theory and Hypothesis Development

### Expertise Heterogeneity and Knowledge Integration Capability of the Entrepreneurial Team

The expertise heterogeneity of the entrepreneurial team refers to the degree to which a member of the team has complementary or different knowledge from other members of the team (Zhang et al., 2020). In previous studies, different scholars explained the impact of heterogeneity on team process from different theoretical perspectives, with significantly varied results. The two most common theoretical bases are social categorization theory and information strategy theory. The former suggests that team otherness and diversity hinder team cooperation, which is adverse to the improvement of team human relations (Hong et al., 2019); the latter, based on the information and decision-making theory, holds that knowledge otherness and diversity play a positive role in promoting team information acquisition and new knowledge generation (Leroy et al., 2021). Therefore, it can be assumed according to the information process theory that the cognition and skills heterogeneity among team members will promote more comprehensive communication and task information sharing within the team, thereby improving the effectiveness of team information and knowledge processing.

In the existing studies related to entrepreneurial team, most scholars hold the view that entrepreneurial teams are in a turbulent, complicated and changeable entrepreneurial environment. Given such context, more innovative entrepreneurial decision-making is necessary to cope with the unpredictable environment. In addition, the differentiated and diversified knowledge background featured by team expertise heterogeneity, which essentially means the complementary knowledge of team members, can help improve the innovativeness and effectiveness of entrepreneurial decision-making (Chen, 2019; Zhang et al., 2020). Based on the information processing theory, this paper holds that the expertise heterogeneity of the entrepreneurial team can provide the team with more abundant and comprehensive accesses to information knowledge. Multidimensional knowledge perspective will in turn improve the knowledge acquisition ability of the team, expand the team horizon, and enable the team to explore from different knowledge perspectives, thereby allowing it to take full advantage of the broad views of its members (Cunningham, 2015) to enhance its ability of knowledge acquisition and integration. Therefore, our paper proposes that the expertise heterogeneity of a team plays a critical role in the enhancement of its integration capability.

***Hypothesis 1:*** *Expertise heterogeneity of an entrepreneurial team is positively correlated with its knowledge integration capability*.

### The Moderating Effect of Team Reflexivity

Team reflexivity has been proved to be capable of helping team members to better understand the expectations on each team member, and develop new understandings and methods to deal with the challenges faced by the team (Wang et al., 2021). Previous studies show that team reflexivity is usually associated with effective team processes, such as promoting team innovation and leadership development (Chen et al., 2018). In entrepreneurial teams, especially in the team with high expertise heterogeneity, a high degree of reflexivity often means higher possibility for the team members to accept different views and opinions. These team members are less likely to regard the differing perspectives or viewpoints between them as a threat adverse to them; instead, they are more likely to regard those views as sincere opinions (Shin et al., 2016). Therefore, this paper argues that team reflexivity can improve the relationship between the expertise heterogeneity of entrepreneurial team and team knowledge integration. Higher reflective entrepreneurial team members are prone to put forward unique perspectives in the process of team information integration, and analyze the differences in the viewpoints among different members in a more rational manner (Schmutz et al., 2018), thereby fully integrating the knowledge perspectives of the members. Meanwhile, team reflexivity is conducive to reducing the situation of “one-man show” in the team. Team members are more willing to focus on the task itself and conduct more equal communications, which is conducive to the diversified expressions and exchanges of knowledge information (van Ginkel et al., 2009; Deng et al., 2021). Therefore, team reflexivity can help the entrepreneurial team with high heterogeneity collect knowledge and views more comprehensively and accurately, while improving its knowledge integration ability. Hence, we propose:

***Hypothesis 2*:** *Team reflexivity moderates the relationship between expertise heterogeneity and knowledge integration in entrepreneurial teams, and the relationship is more positive when team reflexivity is high (rather than low)*.

### The Relationship Between Knowledge Integration and Entrepreneurial Decision of Entrepreneurial Team

Previous literature showed that more extensive knowledge and information can often be obtained if a team can effectively integrate and use unique knowledge resources of its members, thereby improving its decision-making quality and achievements (Zhang et al., 2020; Wang et al., 2021). Moreover, team knowledge integration is considered to be the most important factor affecting team performance (Burmeister et al., 2019). Entrepreneurial team is composed of several members with different knowledge backgrounds, therefore bringing different knowledge resources to the team. On the one hand, the integration of knowledge resources can bring different views to the decision-making process of the team, therefore expanding and enriching the overall knowledge pool of the entrepreneurial team (Gardner et al., 2012); on the other hand, effective knowledge integration within the team will encourage team members to attach greater importance to learning from each other, thereby strengthening inner-team communications and cooperation, stimulating innovative and novel team management ideas, and improving the decision-making ability of the team (Feng and Chen, 2020; Zhang and Wang, 2020). Therefore, we propose that higher knowledge integration ability of a team can help improve its information processing effectiveness, thereby raising the quality of entrepreneurial decision-making.

***Hypothesis 3:*** *The knowledge integration ability of an entrepreneurial team is positively related to entrepreneurial decision-making*.

### Moderated Mediating Model

**Figure 1:**
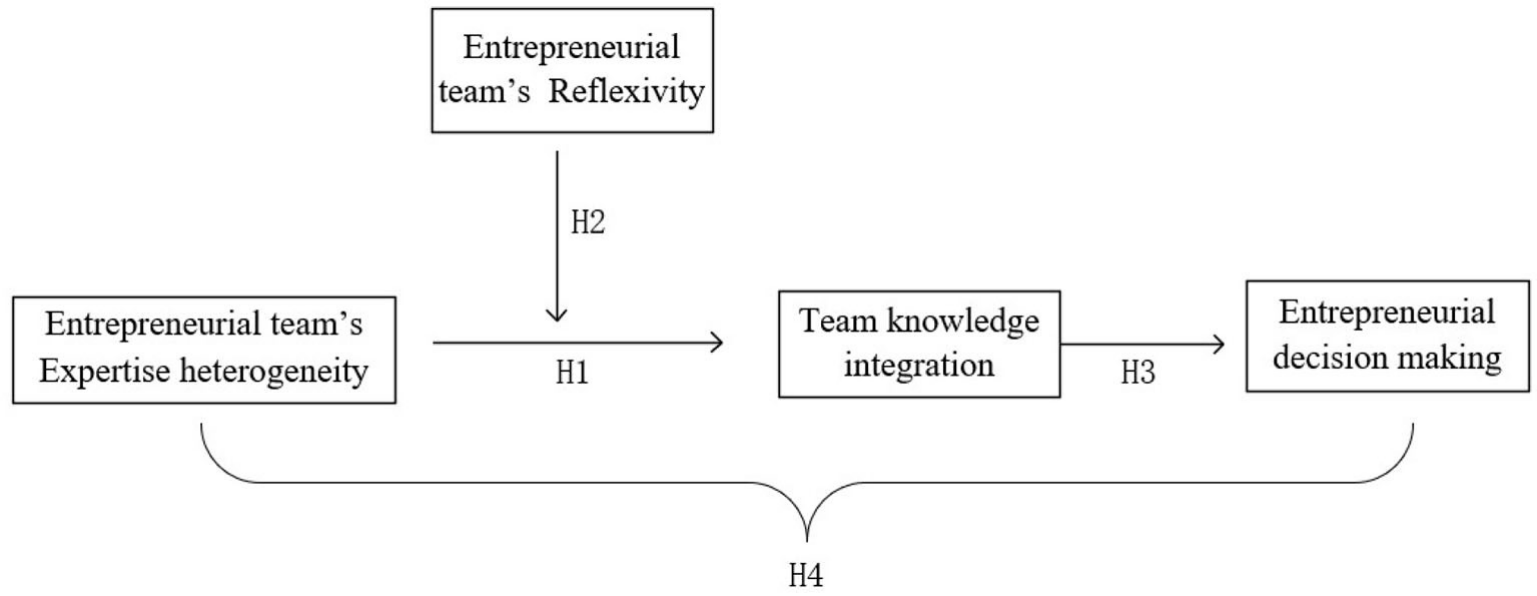
The hypothesized model.

This paper suggests that the knowledge integration ability of an entrepreneurial team is a key mechanism through which the expertise heterogeneity of the team will be able to promote the process of entrepreneurial decision-making. To this end, this paper proposes that the expertise heterogeneity of an entrepreneurial team can positively affect team knowledge integration (Hypothesis 1); team reflexivity can moderate the relationship between entrepreneurial team expertise heterogeneity and knowledge integration (Hypothesis 2); and the knowledge integration ability of an entrepreneurial team exerts a positive impact on entrepreneurial decision-making (Hypothesis 3). Based on the above hypotheses, this paper further proposes a moderated mediating model (Muller et al., 2005; Fan, 2018). To be specific, the expertise heterogeneity of entrepreneurial teams can effectively improve entrepreneurial decision-making. In the context of high team reflexivity, an entrepreneurial team with high expertise heterogeneity is more likely to fully integrate and utilize differentiated knowledge and information of the team members. Such an entrepreneurial team can more easily make effective entrepreneurial decisions when facing market fluctuations. Therefore, we propose (Figure 1 summarizes our overall theoretical model):

***Hypothesis 4:*** *Entrepreneurial team reflexivity moderates the knowledge heterogeneity of an entrepreneurial team through the indirect effect of knowledge integration on entrepreneurial decision-making. In addition, higher team reflexivity will lead to more positive indirect effect*.

## Research Methods

### Sample and Procedure

Our data were collected from the University Science and Technology Park in Zhejiang Province. The college entrepreneurs mainly come from Hangzhou, Ningbo, and Wenzhou. This research is supported by the office director of Zhejiang University Science and Technology Park. According to our preliminary interviews, this sample is particularly applicable for testing our research model. The reasons are mainly threefold. First, these academic entrepreneurial teams are typical knowledge-based entrepreneurial teams, with each team member having its own unique professional knowledge background. This means that such entrepreneurial teams have plenty of talents with different professional skills, which provide us with an excellent context for studying knowledge heterogeneous teams. Furthermore, academic entrepreneurial projects are highly professional and technological, in which team members often work interdependently in the current entrepreneurial surroundings. The present situation calls for more comprehensive integration of different knowledge and insights of team members from diverse field, and turning knowledge integration into a crucial factor for entrepreneurial decision-making. Finally, entrepreneurial projects in University Science and Technology Park are highly innovative. Team members usually hold regular meetings to review and optimize entrepreneurial routines, allowing us to capture the unique role of team reflexivity.

To reduce the concerns about common method bias, a multi-source design was adopted (Podsakoff et al., 2012). A two-wave design with 2-month interval was applied, with a 10-yuan cash reward for the completion of each questionnaire to increase the participation rate among college teachers and students. Data were collected with paper questionnaires to guarantee the confidentiality of their responses. At time T1, 633 questionnaires were collected, among which 566 questionnaires were remained (with an effective rate of 89.41%) after the invalid ones were removed. At time T2, 521 questionnaires were collected, among which 445 were valid (with an effective rate of 85.41%). After all the data were collected, the two-wave response was matched to the interviewees by using the last six digits of their phone numbers, and those data that could not be paired were eliminated. At last, a total of 419 valid questionnaires were obtained in this study. Among the valid samples, 54.1% were men, and 45.9% were women, with an average age of 40–49 years. With respect to the educational level, the samples with college degree, bachelor's degree, master's degree, or above accounted for 25.6, 57.1, or 16.7%, respectively.

### Measures

Expertise heterogeneity of the entrepreneurial team: The 3-item scale suggested by Tiwana and Mclean (2005) was used to assess the team expertise heterogeneity. The Likert-type 7-point scale was adopted, with 1–7 points representing “very disagree” to “very agree,” and with α = 0.90.

Knowledge integration of the entrepreneurial team: The 4-item scale suggested by Tiwana and Mclean (2005) was used to assess the team knowledge integration. The Likert-type 7-point scale was applied, with 1–7 points representing “very disagree” to “very agree,” and with α = 0.88.

Team reflexivity: The 5-item scale suggested by De Jong and Elfring (2010) was applied to measure the team reflexivity. The Likert-type 7-point scale was also used, with 1–7 points representing “very disagree” to “very agree,” and with α = 0.84.

Entrepreneurial decision-making: The 5-item scale suggested by GuiLan (2013) was used to measure the decision-making of the team. The Likert-type 7-point scale was adopted, with 1–7 points representing “very disagree” to “very agree,” with α = 0.92. See Appendix for the specific measurement of the main variables.

Control variables: In addition, variables including gender, age, education background, years of working, and team size were controlled, as these demographic variables were considered to be influential to the integration of entrepreneurial decision-making and entrepreneurial knowledge integration (Hackman and Wageman, 2005).

Analytical approach: We used SPSS19.0 (Norman H. Nie, Dale H. Bent, C. Hadlai Hull) and Mplus7.0 (Muthén & Muthén) for data analysis. The non-standardized regression coefficients were reported below.

## Results

### Confirmatory Factor Analysis

We used Mplus7.4 to conduct a confirmatory factor analysis for examining the discriminant validity of the expertise heterogeneity, entrepreneurial team reflexivity, team knowledge integration, and entrepreneurial decision-making of the entrepreneurial team. The results showed that the model is highly fit well (with χ^2^/df = 1.85, df = 224, RMSEA = 0.05, CFI = 0.96, and TLI = 0.96). Meanwhile, the assumed four-factor model exhibited high fitting degree, significantly superior to the alternative three-factor, two-factor, and one-factor models (see Table 1).

**Table 1:** Comparison of measurement model.

**Model**	**χ^**2**^/df**	**df**	**RMSEA**	**CFI**	**TLI**
One-factor model	10.90	230	0.18	0.53	0.48
Two-factor model	6.11	229	0.13	0.76	0.73
Three-factor model	3.50	227	0.09	0.88	0.87
Four-factor model	1.85	224	0.05	0.96	0.96

*RMSEA, root mean square error of approximation; SRMR, standardized root mean square residual; CFI, comparative fit index*.

### Descriptive Statistics Regression Analysis

Table 2 describes the mean, SD, and correlation coefficient of each variable. It can be seen from the table that expertise heterogeneity of entrepreneurial teams is significantly positively correlated with knowledge integration (with *r* = 0.56 and *p* < 0.01), thereby initially verifying the validity of Hypothesis 1. The knowledge integration of entrepreneurial team is significantly positively correlated with entrepreneurial decision (with *r* = 0.43 and *p* < 0.01), thereby initially verifying the validity of Hypothesis 3. Notably, including or excluding of control variables in this analysis did not change study results.

**Table 2:** Means, SD, and correlations.

**Variables**	**M**	**SD**	**1**	**2**	**3**	**4**	**5**	**6**	**7**	**8**	**9**
Gender	1.47	0.49	1								
Age	3.54	1.19	−0.01	1							
Education background	2.41	0.09	0.05	−0.01	1						
Working years	3.29	1.36	v.03	0.62^**^	−0.06	1					
Team scale	3.13	1.35	0.05	0.21	0.05	0.32^*^	1				
Expertise heterogeneity of the team	4.49	0.92	0.06	0.07	0.04	−0.07	0.16^**^	1			
Knowledge integration	5.42	0.93	−0.03	0.10	0.05	0.26^**^	0.13^*^	0.56^**^	1		
Team reflexivity	5.49	0.93	−0.03	0.03	0.09	0.11^*^	0.18^*^	0.55^**^	0.59^**^	1	
Decision-making	3.93	1.01	−0.01	0.12^*^	0.04	0.16^**^	0.13^*^	0.59^**^	0.43^**^	0.51^**^	1

*Two-tailed test; N = 419*.

** *p < 0.01*,

* *p < 0.05*.

### Hypothesis Testing Results

Hierarchical regression was conducted to test the theoretical hypotheses. The results of the regression analysis are shown in Table 3. Hypothesis 1 predicts that the expertise heterogeneity of an entrepreneurial team is positively correlated with team knowledge integration, as shown in M1, in which the regression coefficient of entrepreneurial team heterogeneity to team knowledge integration is significant (β = 0.30, *p* < 0.01), thereby indicating the validity of Hypothesis 1.

**Table 3:** Regression analysis.

**Variables**	**Team knowledge integration**		**Team decision making**
	**M1**	**M2**	**M3**
**Control variables**			
Gender	−0.02 (0.07)	−0.03 (0.07)	−0.07 (0.06)
Age	0.01 (0.08)	0.03 (0.03)	0.03 (0.05)
Education	0.05 (0.08)	0.02 (0.07)	0.22 (0.07)^**^
Working years	0.01 (0.11)^*^	0.03 (0.11)	−0.03 (0.09)
Team size	0.07 (0.16)	0.10 (0.15)	0.13 (0.13)
**Main effect**			
Expertise heterogeneity	0.30 (0.04)^**^	0.22 (0.04)^**^	
Team reflexivity		0.33 (0.15)^*^	
Knowledge integration			0.42 (0.08)^**^
**Moderation variables**			
Expertise heterogeneity × reflexivity		0.11 (0.04)^**^	
*R^2^*	0.23	0.31	0.32
Δ*R*^2^	0.12	0.07	0.07

*N = 419; unstandardized regression coefficients are reported*.

*Values in bold are relevant to tests of hypotheses*.

** *p < 0.01*,

* *p < 0.05*.

**Figure 2:**
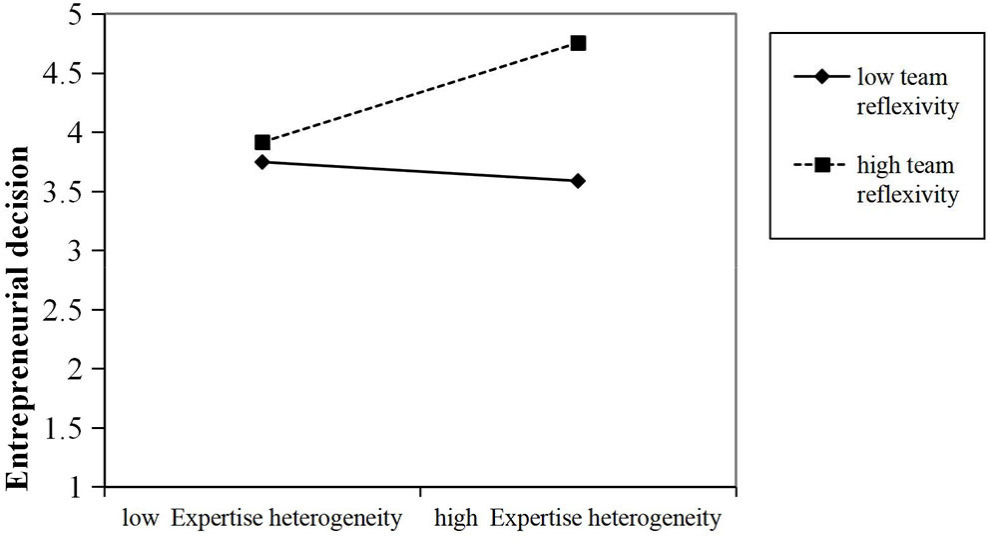
Interaction between expertise heterogeneity and reflexivity on entrepreneurial decision-making.

Hypothesis 2 predicts that the relationship between entrepreneurial team expertise heterogeneity and team knowledge integration is moderated by team reflexivity, as shown in M2. Specifically, when team reflexivity is high, it will also have a more significant moderation effect on the relationship between expertise heterogeneity and knowledge integration of the entrepreneurial team (with β = 0.11, and *p* < 0.01). A simple slope analysis was conducted following the suggestion of Aiken and West (1991) (see Figure 2). As can be seen from Figure 2, within a team with high reflexivity, its expertise heterogeneity is also significantly positively correlated with knowledge integration (1 SD, with β = 0.21, and *p* < 0.001). However, when the team reflexivity is low, the relationship between expertise heterogeneity and team knowledge integration of the entrepreneurial team also becomes less significant (−1 SD, with β = 0.01 and *p* = 0.79). Hence, the assumptions in Hypothesis 2 is supported.

Hypothesis 3 predicts that team knowledge integration can be positively related to entrepreneurial decision-making, just as shown in M3, in which the regression coefficient of team knowledge integration to entrepreneurial decision-making is positively significant (with β = 0.42, and *p* < 0.01), thereby verifying the validity of Hypothesis 3.

Hypothesis 4 further proposes a moderated mediating model: through team knowledge integration, team reflexivity moderates the indirect effect of entrepreneurial team expertise heterogeneity on entrepreneurial decision-making. To further examine the validity of Hypothesis 4, the method recommended by Hayes (2015) was adopted and the indirect effect by Bootstrap analyzed. Mplus7.4 was adopted in this research, and 5,000 repeated samplings was conducted, with the results shown in Table 4. Concretely speaking, the indirect effect of entrepreneurial team expertise heterogeneity on entrepreneurial decision-making is mediated by team knowledge integration, which is more significant with the rise of the team reflexivity, and less significant as the team reflexivity declines. Therefore, the assumptions in Hypothesis 4 is verified.

**Table 4:** Moderated mediating effect.

**Mediating variable**	**Moderated variable**	**Effect**	**Standard error**	**Lower 95%BC CI**	**Upper 95%BC CI**
Entrepreneurial team	High value	0.38	0.03	0.03	0.68
Knowledge integration	Low value	0.06	0.02	−0.10	0.08
	Difference	0.32	0.01	0.13	0.60

*CIs were calculated using the Monte Carlo method with 5,000 repetitions*.

## Discussion

We investigate an important issue in academic entrepreneurial research, that is, the effects of the expertise heterogeneity of entrepreneurial team on entrepreneurial decision-making. Our research findings demonstrate that in an entrepreneurial team, knowledge integration mediates the positive effect of expertise heterogeneity on entrepreneurial decision-making. In addition, it was found that the team reflexivity moderates the relationship between the expertise heterogeneity of the entrepreneurial team and its knowledge integration—the entrepreneurial team with higher reflexivity can make better use of its knowledge resources brought by expertise heterogeneity, which is conductive to the integration of the knowledge resources of the team and the improvement of the entrepreneurial decision level. Ultimately, our findings can provide important implications to the theory, practice, and future research directions in the field of team entrepreneurship.

### Theoretical Contributions

This article contributes to the field of entrepreneurship research by empirically illustrating the efficacy of the knowledge heterogeneity of the entrepreneurial team in improving entrepreneurial decision-making. Specifically, previous studies demonstrating the relationship between entrepreneurial team heterogeneity and entrepreneurial decision-making showed inconsistent conclusions therefore hindering the guidance for management practices. This research examines team heterogeneity from the perspective of knowledge heterogeneity within a team. Based on the team information processing theory, it investigated the mechanism of expertise heterogeneity of the entrepreneurial team on entrepreneurial decision-making and boundary conditions, thereby providing a new perspective for the research of entrepreneurial decision and offering valuable response to the call for deeply exploring the utility boundary of team heterogeneity (Corritore et al., 2019; Nassif, 2019).

Second, our study adds nuances to the relationship between the knowledge heterogeneity and team knowledge integration of the entrepreneurial team. Specifically, the process of how the interplay of the entrepreneurial team knowledge heterogeneity and team reflexivity is related to the team knowledge integration was investigated. Results show that the entrepreneurial team reflexivity determines the extent to which diverse knowledge of the entrepreneurial team can be translated into high-quality entrepreneurial decision-making through team knowledge integration. Accordingly, we highlight the functional role of team reflexivity in entrepreneurial decision-making for entrepreneurial teams with high knowledge heterogeneity.

Finally, we illustrate the underlying mechanism through which the expertise heterogeneity of an entrepreneurial team affects entrepreneurial decision-making. Prior work has investigated how team heterogeneity influences entrepreneurial decision-making *via* the affective process, such as team conflict or team trust (Nikiforou et al., 2018). For instance, members from different backgrounds may spur dysfunctional conflict and induce difficulties in decision-making. Our research pays attention to the intervening process for knowledge integration among team members. With these approaches, we present a new interpretation of how expertise heterogeneity of a team affects entrepreneurial decision-making as a response to the call for a further investigation of the team microprocesses by which team diversity impacts team effectiveness (Lazar et al., 2020).

### Practical Implications

Our findings in this research provide significant enlightenment to entrepreneurial enterprises and managers. First, our results imply that it is beneficial for entrepreneurial teams to extensively absorb team members with different professional knowledge backgrounds (Homan et al., 2020). Complementary perspectives, knowledge, and skills brought by experts from different professional backgrounds can provide access to the non-redundant and diverse information perspective (Salimath and Chandna, 2018), thereby promoting the decision-making of entrepreneurial teams. Managers of entrepreneurial teams should absorb members with diverse knowledge and experience as far as possible. Therefore, it is necessary to recruit team members with different knowledge backgrounds and professional skills in the team-building stage.

Second, our study indicates that it may be necessary for managers to strive to create an environment that facilitates the effective integration of different knowledge and options, while reducing conflict caused by differences in perspectives (Hoever et al., 2018). Our research also suggests that the expertise heterogeneity of an entrepreneurial team can either increase or invalidate its knowledge integration and entrepreneurial decision-making, depending on different levels of team reflexivity. Hence, entrepreneurial teams should encourage their members to express their unique views and opinions bravely, promote team reflexivity (Morris, 2017), enhance the effectiveness of communication, and make efforts to establish an equal, open, and respectful atmosphere for communication (Tost et al., 2013).

Finally, our findings support a relationship between knowledge integration and entrepreneurial decision-making. Our theorizing is that integrating different professional backgrounds is not simply solved by abundant human resources, but rather when a team develops an effective, reliable knowledge communication process. Thus, managers should give special attention to developing the knowledge integration capability with expertise heterogeneity of the entrepreneurial team. Managers may need to carry out more team-building activities to improving team knowledge interaction (Pollack and Matous, 2019).

### Limitations and Directions for Future Research

There are some limitations in this research. First, this study chooses college teachers and students as the research samples, the entrepreneurship by whom is often considered typical of academic entrepreneurship (Agarwal and Shah, 2014). Academic entrepreneurship refers to transforming research-based results from universities, laboratories, or research institutions into commercial products and services by entrepreneurs or entrepreneurial teams (Agarwal and Shah, 2014; Nikiforou et al., 2018). In such type of entrepreneurial teams, the knowledge and skills owned by the team members are the core and critical elements of the entire entrepreneurial team (Agarwal and Shah, 2014). Executing this entrepreneurial decision-making requires integrating expertise and information from different knowledge domains (Lazar et al., 2020). Therefore, the research conclusions of this research may not have referential value to entrepreneurial teams in other industries. For example, the genius of a star designer is more likely to win over the market than a well-integrated team of people in design industries (Berglund et al., 2020). Therefore, future research can be made to expand the territorial and industrial scope, thereby improving the reliability of the research results.

Second, our research merely focuses on knowledge resources inside entrepreneurial teams. However, team members regularly obtain information and knowledge from outside sources (Gardner et al., 2012). In other words, researchers could also examine whether the focal research domain, academic environment networking, their experience in businesses, etc. may lead to any systematic differences in team knowledge integration and entrepreneurial decision-making. For example, IT entrepreneurial teams may differ significantly from biotechnology entrepreneurial teams as IT entrepreneurs tend to move more quickly toward commercialization (Nikiforou et al., 2018). We should acknowledge that this study limits its investigations in expertise resources while overlooking other possible common confounding variables. Future researchers could be conducted to broaden the scope of this research model by including more confounding variables. Such investigation would deepen the understanding of people on how entrepreneurial teams can effectively integrate knowledge resources and achieve high levels of entrepreneurial decision-making.

Third, this research was carried out from the perspective of team information processing. It also takes team reflexivity as boundary conditions for successful team knowledge integration and entrepreneurial decision-making. Future studies can be conducted to test additional moderators from the perspective of college entrepreneurs and business personages. For instance, academic entrepreneurs often lack commercial skills and marketing experience (Visintin and Pittino, 2014). They tend to exhibit superior education, technical, and scientific specialization while having poor performance in industrial experience (Colombo and Piva, 2012). Such entrepreneurial team might achieve high entrepreneurial performance if they had a mix of academic entrepreneurs and experienced business people. Thus, examinations on how the non-academics with a strong business background can be added to the team may bring an utterly different perspective for team knowledge integration and entrepreneurial decision-making.

Finally, this study mainly focuses on the effects of entrepreneurial team expertise heterogeneity on entrepreneurial decision-making. Other entrepreneurial team-level outcomes, such as venture success, more outstanding performance, and higher creativity in entrepreneurial teams, may also be obtained when the teams gain a higher level of expertise heterogeneity and knowledge integration ability (Lazar et al., 2020). For example, this study found that when the knowledge skills and perspectives of team members are fully integrated in an entrepreneurial team, a virtuous cycle of growth and opportunity will also be built within the team. Based on this logic, future research could also be carried out to investigate whether entrepreneurial team expertise heterogeneity can enhance entrepreneurial team effectiveness from other aspects.

## Conclusions

In entrepreneurship research studies, entrepreneurial decision-making is generally considered as a complex process, which plays a decisive role in entrepreneurial results. However, how could an entrepreneurial team harness the knowledge of team members and make better entrepreneurial decision-making? To answer this question, this study examines the effects of entrepreneurial team knowledge heterogeneity on knowledge integration and entrepreneurial decision-making. Furthermore, we found that an entrepreneurial team with different levels of team reflexivity may also apply its knowledge heterogeneity differently. Specifically, a moderated mediation model demonstrates that in an entrepreneurial team with higher reflexivity, the expertise heterogeneity of the entrepreneurial team is more positively related to the entrepreneurial decision-making through knowledge integration. Through this study, it is hoped that it will facilitate further research studies in exploring the influence of entrepreneurial team expertise heterogeneity to entrepreneurial team dynamics and outcomes and, ultimately, its significant role in promoting the understanding of people on the effectiveness of entrepreneurial teams.

## Data Availability Statement

The raw data supporting the conclusions of this article will be made available by the authors, without undue reservation.

## Ethics Statement

The studies involving human participants were reviewed and approved by Zhejiang Gongshang University Ethics Committee. The patients/participants provided their written informed consent to participate in this study. Written informed consent was obtained from the individual(s) for the publication of any potentially identifiable images or data included in this article.

## Author Contributions

SY designed the study and wrote the draft of this article. YX designed the survey and managed the data. BY also collected the data and analyzed the data. DZ participated in the survey adaptation and collected data. All the authors reviewed the draft and contributed to the final version of the manuscript.

## Funding

This work was supported by National Natural Science Foundation of China (No. 72074195) and Project of Philosophy and Social Science Research, Ministry of Education, China (No. 19YJA630092).

## Conflict of Interest

DZ was employed by company Zhejiang Tianyuan Building Material Company. The remaining authors declare that the research was conducted in the absence of any commercial or financial relationships that could be construed as a potential conflict of interest.

## Publisher's Note

All claims expressed in this article are solely those of the authors and do not necessarily represent those of their affiliated organizations, or those of the publisher, the editors and the reviewers. Any product that may be evaluated in this article, or claim that may be made by its manufacturer, is not guaranteed or endorsed by the publisher.

## Appendix

**Table:** Scales and items used in the study.

**Constructs**	**Sources**	**Items**
Entrepreneurial team's expertise heterogeneity	Tiwana and Mclean (2005)	Members of our team vary widely in their areas of expertise.
		Members of our team have a variety of different backgrounds and experiences.
		Members of our team have skills and abilities that complement each other's.
Entrepreneurial team's knowledge integration:	Tiwana and Mclean (2005)	Members of our team synthesize and integrate their individual expertise at the team level.
		Members of our team span several areas of expertise to develop shared team-related concepts.
		Members of our team can clearly see how different pieces of team members fit together.
		Members of our team competently blend new task-related knowledge with what they already know.
Entrepreneurial decision-making	GuiLan (2013)	Before formally discussing the decision, the members of our entrepreneurial team have a clear idea of the decision-making scheme.
		Members of our entrepreneurial team actively express their opinions during the decision-making process.
		After discussion, our team members finally reached an agreement in the process of entrepreneurial decision-making.
		In the process of entrepreneurial decision making, our entrepreneurial team members will fully analyze and evaluate the decision risks.
		You have no regrets about the decisions made by your entrepreneurial team.
Team reflexivity	De Jong and Elfring (2010)	In our team we often review the feasibility of our objectives.
		In our team we often discuss the methods used to get the job done.
		In our team we regularly discuss whether we are working effectively together.
		In our team we modify our objectives in light of changing circumstances.
		In our team we modify our objectives in light of changing circumstances.
		In our team we often review our approach to getting the job done.
